# Image Encryption Using a Spectrally Efficient Halton Logistics Tent (HaLT) Map and DNA Encoding for Secured Image Communication

**DOI:** 10.3390/e24060803

**Published:** 2022-06-08

**Authors:** Sakshi Patel, Thanikaiselvan Veeramalai

**Affiliations:** School of Electronics Engineering, Vellore Institute of Technology (VIT), Vellore 632014, India; sakshi.patel@vit.ac.in

**Keywords:** Halton sequence, chaotic maps, 5D hyper-chaotic system, DNA computation, Lyapunov exponent spectrum, spectral entropy, image encryption

## Abstract

With the advancement of technology worldwide, security is essential for online information and data. This research work proposes a novel image encryption method based on combined chaotic maps, Halton sequence, five-dimension (5D) Hyper-Chaotic System and Deoxyribonucleic Acid (DNA) encoding. Halton sequence is a known low-discrepancy sequence having uniform distribution in space for application in numerical methods. In the proposed work, we derived a new chaotic map (HaLT map) by combining chaotic maps and Halton sequence to scramble images for cryptography applications. First level scrambling was done by using the HaLT map along with a modified quantization unit. In addition, the scrambled image underwent inter- and intra-bit scrambling for enhanced security. Hash values of the original and scrambled image were used for initial conditions to generate a 5D hyper-chaotic map. Since a 5D chaotic map has complex dynamic behavior, it could be used to generate random sequences for image diffusion. Further, DNA level permutation and pixel diffusion was applied. Seven DNA operators, i.e., ADD, SUB, MUL, XOR, XNOR, Right-Shift and Left-Shift, were used for pixel diffusion. The simulation results showed that the proposed image encryption method was fast and provided better encryption compared to ‘state of the art’ techniques. Furthermore, it resisted various attacks.

## 1. Introduction

In the past few decades, use of technology has grown rapidly. Transmission and reception of text, voice, image or video is possible due to growth in the field of communication. Large amounts of data are transferred from one point to another every second, which gives rise to security concerns regarding private information. Digital images are relevant and contain large amounts of information. Therefore, lots of research has been done in order to prevent unauthorized access to malicious nodes. Many techniques, like image encryption, steganography and watermarking, are used to protect images from attackers [1]. Image encryption is a method whereby the input image is converted to an unreadable format, which cannot be decrypted without a key. There are two main methods through which a meaningful image can be converted into an unreadable format: confusion and diffusion. Confusion is a process whereby the position of the pixels is changed in such a manner that, visually, it becomes impossible to predict the original image. On the other hand, diffusion is a process whereby the pixel values are changed to encrypt the image. AES (Advanced Encryption Standard) and DES (Data Encryption Standard) entail some of the very first algorithms designed for cryptography. Due to new advancements in communication systems these methods are now found to be dated, have many defects and are more suitable for text encryption [2]. In order to overcome these problems, modern research has focused more on chaos theory, encoding techniques and artificial intelligence. Chaotic maps are extremely sensitive to initial conditions, and are nonlinear, highly random and unpredictable in nature. These characteristics are used in cryptography applications in order to provide better security in the channel [3,4]. The main motivation of the proposed research work was to prevent multimedia threats in the communication channel. Traditional encryption schemes use chaotic maps to confuse and diffuse the pixels. The initial and control parameters used in these algorithms are fixed throughout the encryption process for all input images; which makes the image vulnerable to attack. Higher dimensional maps show more randomness and complex dynamic behavior, compared to traditional one-dimensional chaotic systems.

In general, image encryption methods contain two phases: image scrambling, using chaotic maps, and pixel diffusion, using XOR operation. Various encryption algorithms have been developed by researchers. In [5], a chaos-based cryptosystem was proposed, composed of several rounds of diffusion and substitution. Chaotic maps were used to shuffle the pixels of the image and values were sequentially altered to achieve diffusion. The pixel confusion algorithms included new sorting methods literature, like the quantization unit [6], matrix semi-tensor product theory [7,8], zigzag permutation, graph-theory and fractal sorting matrix [9], to correctly decrypt the image at the receiver’s end. Authors in [10] proposed a color image encryption scheme, based on one-time keys and chaotic maps. This algorithm generated keys by means of the MD5 algorithm of the mouse position, which makes the algorithm robust to chosen-plaintext attack. Color image encryption algorithm, using multi dimension systems with bit-level permutation, was proposed in [11]. For the scrambling and diffusion process, a piece-wise linear chaotic map and Chen multi-dimensional system were used, respectively. In [12], the initial keys for the chaotic maps were generated by MD5 algorithm with input image pixels. This method ensured a different key for every input image. The research discussed above were based on the traditional one-dimensional chaotic map, which is also well known to hackers. Therefore, there is a need to design a new robust chaotic map and an efficient encryption algorithm for secured communication. Multidimensional chaotic maps are a new subject of research as they provide better hyper-chaotic behavior than the one-dimensional chaos system. In 1963, the first chaotic attractor was found which led to development of chaos theory in many fields [13]. The hyper-chaotic system enhances randomness and indefiniteness; therefore, it is more popular in engineering applications. In [14], a 5D hyper-chaotic map was used to generate pseudorandom sequences. The obtained sequences were recombined for confusion and diffusion of image pixels. The two popular hyper-chaotic systems are Rössler [15] and Chua’s circuit [16]. Chen’s hyper-chaotic system [17] is also a popular technique to generate pseudorandom sequences and it is widely used by researchers for image encryption [18]. Authors in [19] used a 5Dhyper-chaotic system for secure communication, based on a microcontroller unit. New multidimensional chaotic systems were obtained in order to achieve better randomness and complex dynamical behavior [20,21,22,23]. High-dimensional systems, combined with neural networks, also provide high security and resistance against various attacks [24]. Recent research has been based on compressive sensing, where the input image is compressed to a smaller size which is, then, further embedded with secret data and encrypted to achieve highly secured image communication [25]. This method has an extra advantage of reserving less bandwidth while the data is transmitted to the receiver. Mathematical characteristics of chaotic attractors are rigorously studied. Sequences like the Halton sequence [26], which shows uniform behavior in space, cannot be used in cryptography. These sequences need to be scrambled to obtain non-uniform behavior for security applications. Research in [27], evaluated some pre-existing scrambling techniques of the Halton sequence and also proposed simple techniques, like increasing the number of points between the bases, in order to generate a random Halton sequence. In [28], a survey of randomized quasi-Monte Carlo methods was conducted to study the transformation of uniformly distributed sequences after applying scrambling methods. For better security, bit-level operators were applied on the shuffled image [29,30]. DNA-based pixel diffusion algorithms mainly focus on changing the gray pixel value to DNA base streams. Further, various DNA operations, like compliment or XOR, were applied among the base values to diffuse the pixels. Finally, the pixels were converted back to gray level values. DNA-fused chaos theory is a very popular and successful method for image pixel diffusion. These approaches are very sensitive to initial conditions and are resistant to various brute attacks. DNA-based encryption technique is blended with chaotic maps, hash functions and other methods to provide better security [31,32,33,34]. Authors in [35] generated random sequence, using coupled map lattice and chaotic map, which are then combined with the DNA method for image encryption. DNA encoding was also used for medical image encryption combined with chaotic maps in the frequency domain to achieve robustness in the proposed algorithm [36,37,38,39,40,41].

The proposed work focused on building a new chaotic map (HaLT map) by combining Logistic Tent map and the Halton sequence. This map can be used in many engineering applications where a system with high chaotic nature is required. In the proposed algorithm, a new HaLT map was used in multimedia security applications to provide better privacy from unauthorized users. In this work, two levels of scrambling process were applied to achieve high randomness among image pixels. The use of a HaLT map and inter–intra bit-level permutation was done to confuse the pixels in the first and second levels of the scrambling process, respectively. Further, a 5D hyper-chaotic map and DNA computations with seven operations were used in the diffusion stage. The initial and control parameters for the 5D chaotic map were generated using MD5 and SHA256 hash functions. The initial seed to the hash functions were pixels of the input image. This method generated a new key for every input image, making the algorithm resistant to cryptography attacks.

The main contributions of the proposed scheme are as follows,

➢ A novel random sequence (HaLT map) generator is proposed, which combines a CLT map (Combined Logistic Tent map) and the Halton sequence.
➢ A modified quantization unit is developed to sort the generated HaLT sequence for first level scrambling.
➢ For second level scrambling, bit-level operations are performed for enhanced security.
➢ MD5 and SHA256 hash functions are obtained from an original and scrambled image, respectively, and they are used to calculate the initial seeds for a 5D hyper-chaotic map.
➢ A five-dimension chaotic map is used for DNA computing in order to provide great confidentiality and high security.
➢ Pixel permutation is performed by double sorting in the quantization unit to efficiently change the pixel position of the matrix.
➢ Seven DNA operations, namely ADD, SUB, MUL, XOR, XNOR, Right-Shift and Left-Shift, are performed to efficiently diffuse the pixels of the permutated image.
➢ The selection of DNA rules and seven operations are carried out using the five chaotic sequences obtained from the 5D hyper-chaotic map.

Further organization of this research paper is as follows; preliminaries of the proposed methodology is described in Section 2; step by step explanation of the proposed methodology is given in Section 3; Section 4 discusses the results obtained and the conclusion is given in Section 5.

## 2. Preliminaries

### 2.1. Halton Sequence

In statistics, Halton sequence is a standard low-discrepancy sequence which seems random but is deterministic in nature. This sequence generates points in space which cover the domain uniformly [26]. It is very popular among researchers because of its ease of implementation, due to its definition via the radical inverse function. Halton sequence is highly correlated between the inverse function and the base used for different dimensions, which results in poor randomization of the points in space. This sequence is generated using co-prime numbers as its base. If q is an integer, then it can be expressed in terms of base b as shown in Equation (1),

(1) $$q=\overset{k}{\underset{i=0}{\sum }}d_{i}b^{i}$$

where, $d_{i}$ is the sequence of digits $d_{i}\ldots \;d_{2}d_{1}$, k is the number of points. The $q^{th}$ number in the Halton sequence is given in radical inverse of Equation (2), where all the digits $d_{i}$ are in the interval 0 to 1.

(2) $$H\left(q,b\right)=\overset{k}{\underset{i=0}{\sum }}d_{i}b^{i-1}$$

Consider, base b=2 the interval 0 to 1 is divided into half, then into a fourth, then eighth, and so on. Notice that the Halton sequence is basically filling the gaps between the intervals. This behavior is similar to uniform distribution. Therefore, to overcome this phenomenon, Halton sequences are randomized. Instead of using a traditional chaotic method for image scrambling, we proposed an algorithm to scramble Halton sequences using combined chaotic map to obtain a nondeterministic sequence for better pixel scrambling for image encryption applications.

### 2.2. Cryptographic Hashing

A cryptographic hash function is an algorithm that converts input data (message) into a fixed length of bit array (hash, digest, hash value). This function has many information security applications as it is able to withstand all known cryptanalytic attacks [1]. Using a dedicated algorithm [10,12,25,41], any input data can be converted to a secret hash value. The two hash functions used in the proposed work are:

#### 2.2.1. MD5

Message Digest 5 is a hash function which gives 128 bits of hash value. It has a one-way function which converts any input data to fixed length, hexadecimal, output bits, in order to authenticate the original message.

#### 2.2.2. SHA256

Secure Hash Algorithm-256 is a modified version of the MD5 algorithm, providing more security and authenticity to the original data. The hash value obtained by SHA can take years or decades to break, thus making it unbreakable. Another advantage of this function is its uniqueness, i.e., there are likely to be few, or no, collisions between the two hash values.

The detailed explanation of how these hash functions were used in the proposed work is discussed in Section 3.1.3.

### 2.3. Chaotic Maps

Chaotic maps are mathematical functions that exhibit chaotic behavior. These maps are used for generating random sequences for various engineering applications [6]. The two chaotic maps used in the proposed algorithm are:

#### 2.3.1. Logistic Map

**Figure 1:**
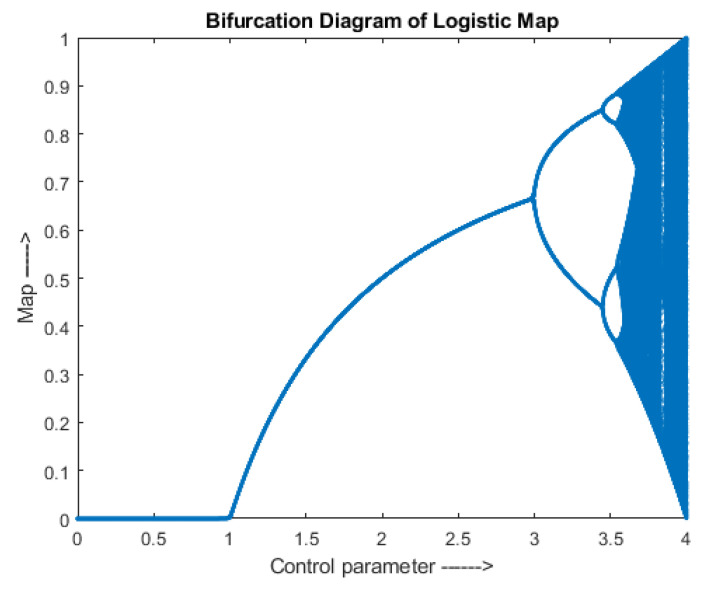
Logistic Map-Bifurcation Diagram.

A logistic map is a member of the chaos family, represented in Equation (3),

(3) $$p_{n+1}=\mu *p_{n}*\left(1-p_{n}\right)$$

where, *µ* is a positive number with range (3.5, 4). It is also known as biotic potential, which has a maximum capacity to generate chaotic values. $p_{n+1}$ and $p_{n}$ are the outputs in the range (0, 1) for ${\left(n+1\right)}^{th}$ and $n^{th}$ iteration, respectively. A bifurcation diagram of the logistic map is shown in Figure 1.

#### 2.3.2. Tent Map

**Figure 2:**
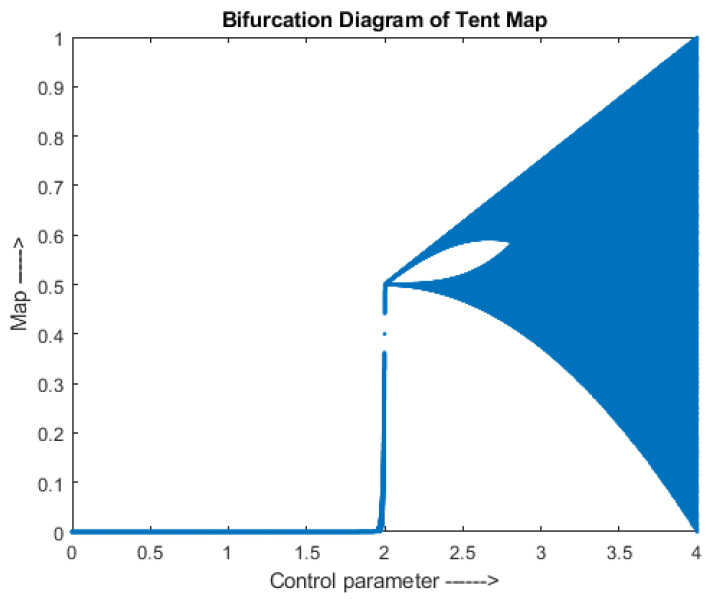
Tent Map-Bifurcation Diagram.

A tent map is a member of the chaos family, represented in Equation (4),

(4) $$p_{n+1}=\left\{\begin{array}{c} \frac{\alpha *p_{n}}{2};for\;p_{n}<0.5\\ \frac{\alpha *\left(1-p_{n}\right)}{2};for\;p_{n}\geq 0.5 \end{array}\right.$$

where, α is a positive number with range (2, 4), $p_{n+1}$ and $p_{n}$ are the outputs in the range (0, 1) for ${\left(n+1\right)}^{th}$ and $n^{th}$ iteration, respectively. A bifurcation diagram of a tent map is given in Figure 2.

#### 2.3.3. Combined Chaotic Map: Combined Logistic-Tent (CLT) Map

The combined maps are obtained using Equation (5),

(5) $$p_{n+1}=mod\left(X\left(x_{1},y_{1}\right)+Y\left(x_{2},y_{2}\right),1\right)$$

where, X and Y are the two maps to be combined, x is the initial value and y is the control parameter of the maps. $p_{n+1}$ gives outputs in the range (0, 1) for ${\left(n+1\right)}^{th}$ iteration. A combined Logistic-Tent map was generated as per Equation (5) and it is represented in Equation (6), where X is the logistic map, Y is the tent map, $\left(x_{1},x_{2}\right)$ = ($p_{n}$,$p_{n}$) are the initial values of the logistic and tent maps, respectively, and $\left(y_{1},y_{2}\right)$ = (μ,α) are the control parameters of the logistic and tent maps.

(6) $$p_{n+1}=\left\{\begin{array}{c} mod\left(\mu *p_{n}*\left(1-p_{n}\right)+\frac{\alpha *p_{n}}{2},1\right);for\;p_{n}<0.5\\ mod(\mu *p_{n}*\left(1-p_{n}\right)+\frac{\alpha *(1-p_{n})}{2},1);for\;p_{n}\geq 0.5 \end{array}\right.$$

**Figure 3:**
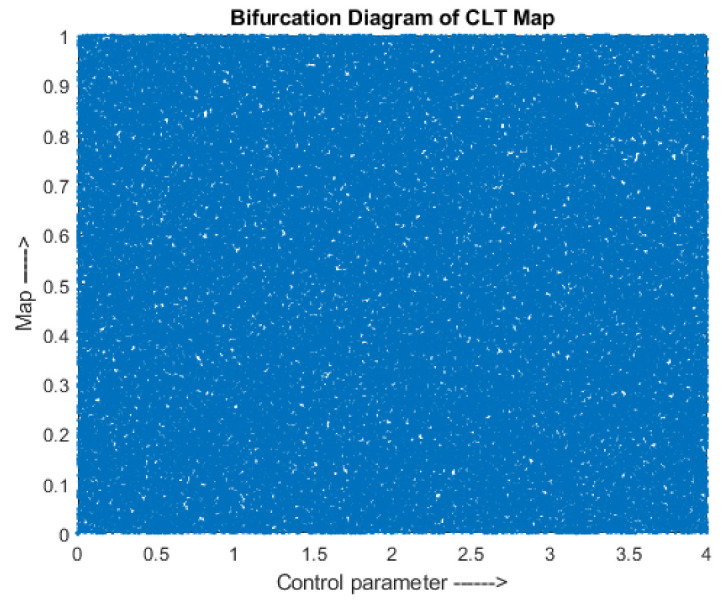
Combined Logistic Tent map—Bifurcation diagram.

μ and α are in the range 3.5 to 4 and 2 to 4, respectively. The outputs $p_{n+1}$ and $p_{n}$ are in the range (0, 1) for ${\left(n+1\right)}^{th}$ and $n^{th}$ iteration, respectively. The bifurcation diagram is shown in Figure 3 which verifies that the map is chaotic in the range (0, 4).

#### 2.3.4. Hyper-Chaos System: 5D Hyper-Chaotic Map

**Figure 4:**
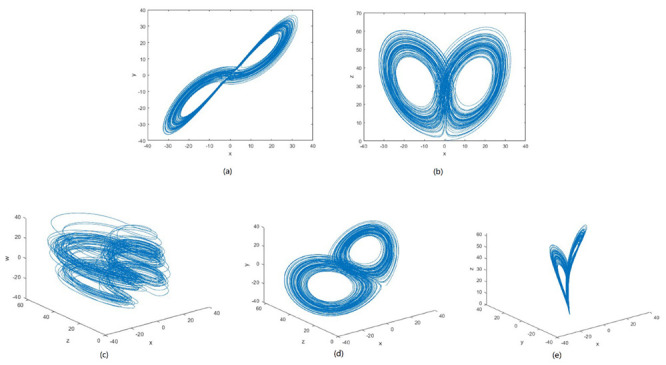
Phase diagram of 5D Hyper−chaotic system with parameters ($b_{1}$, $b_{2}$, $b_{3}$, $b_{4}$, $b_{5}$, $b_{6}$, $b_{7}$, $b_{8}$ ) = (35, 7, 35, −5, 10.6, 1, 5, 0.05). (**a**,**b**) 2D projection $a_{1}\;$−$\;a_{2}$, $a_{1}-a_{3}$ respectively. (**c**–**e**) 3D projection $a_{1}$ − $a_{3}$ − $a_{5}$, $a_{1}$ − $a_{3}$ − $a_{2}$, $a_{1}$ − $a_{3}$ − $a_{2}$ respectively.

Chaos theory is used in many fields, such as secured communication and mathematics. The logistic map discussed above is a one-dimensional chaotic map, used to generate random sequences for various applications. When hyper-chaotic systems are compared with lower dimension chaotic systems, they provide strong confidentiality, high randomness, more complex dynamic behavior, large key space and unpredictability with at least two positive Lyapunov exponents [22]. For any n-dimensional Hyper-Chaotic map consisting of n−2 Lyapunov exponents, the following two conditions should be satisfied: firstly, the phase space, where the Hyper-Chaotic map exists, should be at least n, which means the number of coupled first-order differential equations required are n. Secondly, at least two terms should be present in the differential equations which provide dynamic instability, and at least one should be nonlinear in nature. In the proposed research, the following 5D Hyper-Chaotic attractor was used, which is shown in Equation (7). This system was derived from a 3D modified Lorenz system, by adding a coupling and a nonlinear feedback controller, which gave rise to two quadratic nonlinearities.

(7) $$\left\{\begin{array}{c} \dot{a_{1}}=b_{1}\left(a_{2}-a_{1}\right)\\ \dot{a_{2}}=b_{3}a_{1}+b_{4}a_{2}-a_{1}a_{3}+a_{3}\\ \dot{a_{3}}=-b_{2}a_{3}+a_{1}{}^{2}\\ \dot{a_{4}}=b_{5}a_{2}+b_{6}a_{4}\\ \dot{a_{5}}=-b_{7}a_{1}-b_{8}a_{5} \end{array}\right.$$

where, $b_{1}$, $b_{2}$ > 0, $b_{3}$ > −$b_{4}$, $b_{5}b_{7}$ ≠ 0, $b_{1}$, $b_{2}$, $b_{3}$, $b_{4}$ and $b_{6}$ are constant parameters, $b_{5}$ is the coupling coefficient and $b_{7}$, $b_{8}$ are control parameters. The 3D and 2D projection of the 5D hyper-chaotic system is shown in Figure 4.

### 2.4. DNA Computing for Cryptography

Genetic code in a living cell is a term used for the set of instructions that translates DNA information within genetic material into amino acid sequences of proteins. This encoding technique is a vast area of research, not only in the field of biology, but also in other branches, like science and engineering. A DNA sequence contains four types of proteins: namely, Adenine (A), Thymine (T), Cytosine (C), Guanine (G). The bases A and T are complementary, and G and C are complementary to each other. Similarly, in the binary system, 1 and 0 are complementary; therefore, 00 and 11 are also complementary, as are 10 and 01complementary to each other. Using DNA to encode a binary system 00, 11, 10 and 01, there are 4! = 24 combinations possible. Among these 24 combinations, only 8 kinds of bases satisfy the complementary rule, shown in Table 1 [38,39].

**Figure 5:**
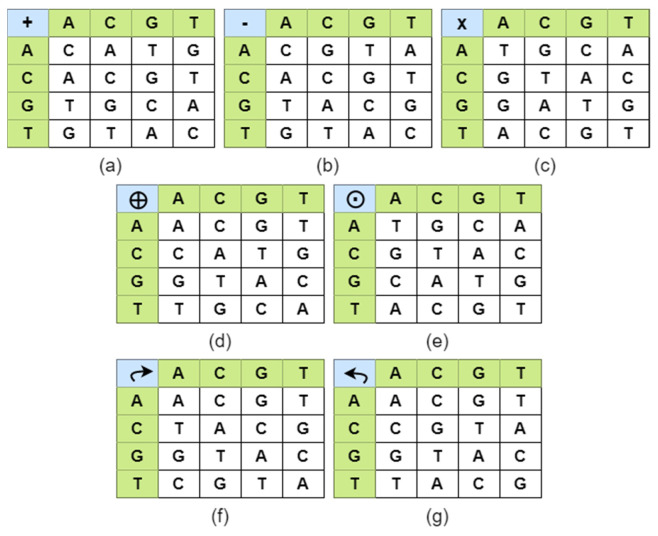
DNA algebraic operations (**a**) ADD, (**b**) SUB, (**c**) MUL, (**d**) XOR, (**e**) XNOR, (**f**) Right circular shift, (**g**) Left circular shift.

Randomly, a rule is chosen among eight rules in order to DNA encode image pixels. For example, consider a pixel value 156 in decimal is converted to binary value ‘10011100’. This binary sequence can be encoded to 8 kinds of amino acid strands, ‘GCTA’, ‘CGTA’, ’GCAT’, ‘CGAT’, ‘TAGC’, ‘ATGC’, ‘TACG’, ‘ATCG’, using the rules in Table 1. Among these 8 DNA sequences, any one rule is chosen for a particular pixel value. The seven possible DNA operations used in this work are: ADD, SUB, MUL, XOR, XNOR, Right Circular Shift and Left Circular Shift. These operations were derived based on fusing mathematics and biological operators, which are shown in Figure 5. The seven DNA operations were performed according to the binary ADD, SUB, MUL, XOR, XNOR operations. In right and left circular shift, the binary value of the image pixels was circularly shifted and the DNA encoded according to the rules given in Figure 5f,g. These operations were used to fuse the plain image pixels with key image pixels. In order to provide good security to the input images, a different operation was used for every pixel value.

## 3. Proposed Image Encryption Methodology

**Figure 6:**
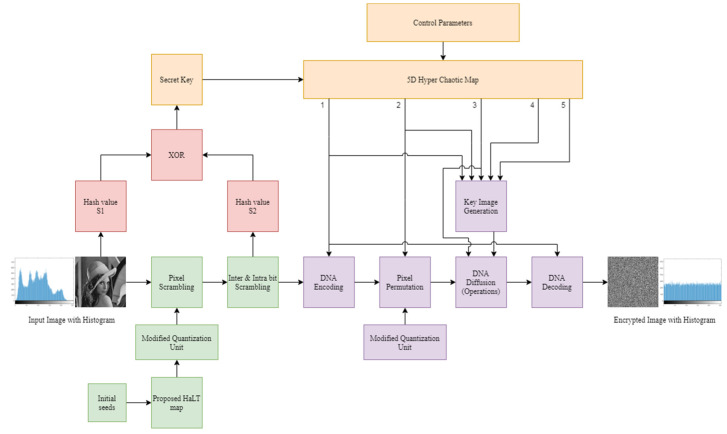
Block Diagram of Proposed Image Encryption Method; 1–5 are the five sequences of 5D Hyper−chaotic map.

The proposed image encryption methodology consisted of two phases which are explained in the following subsections. Figure 6 gives the proposed block diagram.

### 3.1. Phase 1

The encryption steps in this phase contain first level scrambling, with the proposed HaLT map, second level scrambling with bit-level operations, and seed generation, using hash functions for the diffusion process.

#### 3.1.1. Proposed HaLT Map

As discussed earlier, Halton sequences are deterministic in nature and show uniform distribution in space. Therefore, there is a need to scramble the Halton sequence to use it in information security applications. In this research, the CLT chaotic map was used to efficiently scramble the Halton sequence.

**Figure 7:**
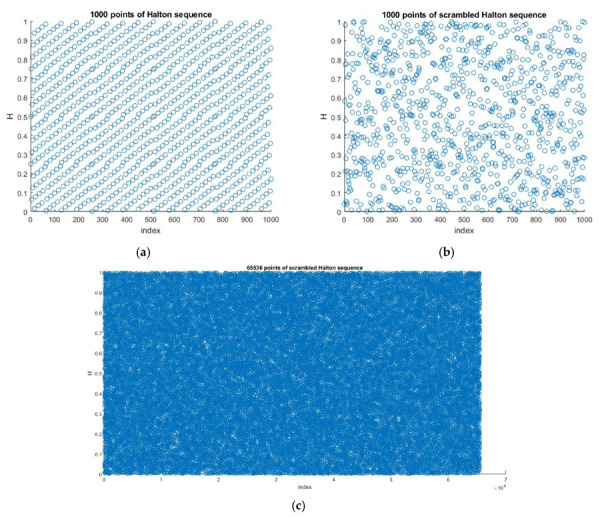
(**a**) 1000 Halton sequence points for base = 4, (**b**) 1000 HaLT map points, (**c**) 65,536 HaLT map points.

The idea behind combining any two chaotic maps is given in Equation (5) and it is used in this method. Consider a Halton sequence obtained using Equation (2) with base b=4, the 1000 points generated are given in Figure 7a. To generate a scrambled sequence, the combined chaotic-CLT map was used. As discussed earlier, the CLT map was obtained by combining logistic and tent chaotic maps, which showed high randomness in the range 0 to 1. Similarly, using Equation (5), the Halton sequence and CLT map were combined to get a new HaLT map for input image scrambling. The 1000 random points of the HaLT map in the interval 0 to 1 are shown in Figure 7b. Figure 7c shows 65,536 random points of the generated map for the application in [256 × 256] size image encryption.

**Figure 8:**
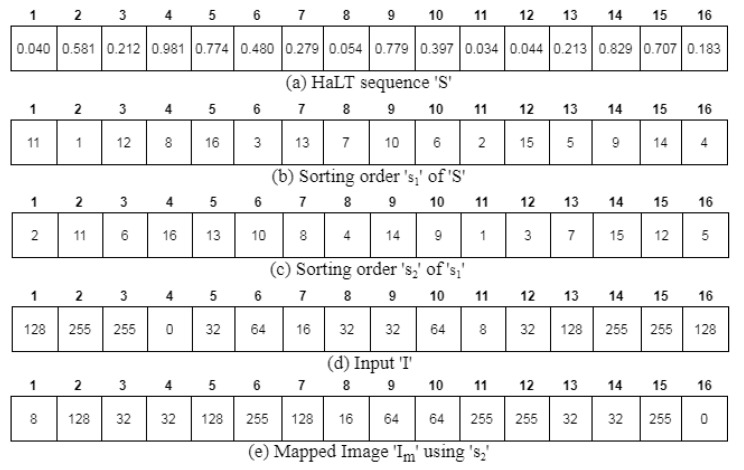
Modified Quantization Unit; 1–16 are the number of pixels.

In [6], the quantization unit was used for pixel scrambling, where the input image was mapped with the sorted chaotic map. Here, the HaLT map was double sorted, which was then mapped with the input image. Consider the generated HaLT sequence S shown in Figure 8a. This sequence S was sorted and the sorting order $s_{1}$ recorded as shown in Figure 8b. In the modified quantization unit, $s_{1}$ was arranged in ascending order to obtain $s_{2},$ as shown in Figure 8c. This sorted sequence was used to map the input image pixels I given in Figure 8d. Finally, the first level of scrambled image $I_{m}$ was obtained, as shown in Figure 8e.

#### 3.1.2. Bit-Level Operations

**Figure 9:**
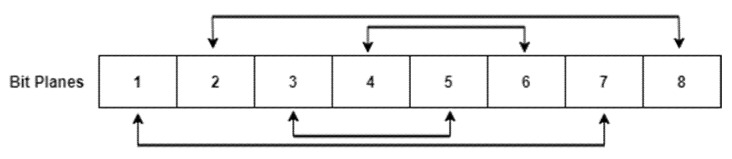
Intra-bit scrambling; 1–8 are the eight planes of the image.

The next step in the first phase of the proposed methodology is inter- and intra-bit scrambling. The proposed bit scrambling algorithm scrambles the bits of the image pixels and the planes of the image. After the first level of image scrambling, bit plane slicing was done to extract the eight planes of the matrix. Now the odd and even number of planes were swapped among each other, as shown in Figure 9. In inter-bit scrambling, the even bit plane pixels were flipped up to down, using Equation (8), where $arr_{new}$ and $arr_{old}$ were the new and old bit plane arrays, respectively, flipud is the function in MATLAB which flips the array up to down, and i is the index for bit plane varying from 1 to 8. The image arrays were then converted from binary to decimal values and reshaped to the size of the original image to obtain a final scrambled image $I_{s}$.

(8) $$arr_{new\left(i\right)}=\left\{\begin{array}{c} flipud\left(arr_{old\left(i\right)}\right)\;\;\;\;\;\;\;if\;\;\;\;\;\;\;\;arr_{old\left(i\right)}=even\\ arr_{old\left(i\right)}\;\;\;\;\;\;\;\;\;\;\;\;\;\;\;\;\;\;\;\;\;if\;\;\;\;\;\;\;\;arr_{old\left(i\right)}=odd \end{array}\right.\;$$

#### 3.1.3. Seed Generation Using Hash Functions

The last step of the first phase was to generate initial seeds for the 5D hyper-chaotic map. In this method, SHA-256 and MD5 hash functions were used. Two hashes were obtained, one from the original image and another from the scrambled image, which were then XOR to create the seed for the chaotic map. Hash functions play a vital role in cryptography, because they are irreversible in nature and they can resist many attacks. Since SHA-256 and MD5 can generate 256 and 128 bits, respectively, they were used in the proposed algorithm to provide better security and prevent various attacks. The number of rows and columns of the original image were considered as $\left[row\times col\right]$.

A pixel value at a particular location can be represented as $I\left(i,j\right)$, where I is the original image and $\left(i,j\right)$ is the $i^{th}$ row and $j^{th}$ column. Three vectors $V_{1}$ of size row, $V_{2}$ of size col and $V_{3}$ of size [row+col−1] were generated from the original image. Here $V_{1}\left(i\right)$ was the sum of all the pixels in the $i^{th}$ row, $V_{2}\left(j\right)$ was the sum of all the pixels in the $j^{th}$ column and $V_{3}\left(i\right)$ was the sum of all the pixels across $i^{th}$ diagonal of image I. The MD5 algorithm was applied on all the three vectors using Equation (9), and then SHA-256 function was applied on the resultant hash value obtained from the previous MD5 algorithm to get a 32-bit hash value, using Equation (10). The above hash value generation algorithm was also applied on the scrambled image $I_{s}$ to get another 32-bit value. The two hash values obtained were further XOR, as given in Equation (11); the resultant initial seed was used as initial seed for the Hyper-Chaotic map in the next phase, where, $M_{i}^{1}$ and $M_{i}^{2}$ are the MD5 hash values obtained from the original image and the scrambled image, respectively. Then, i=1,2,3. $S_{1}$ and $S_{2}$ were the SHA-256 hash values obtained from $M_{i}^{1}$ and $M_{i}^{2}$ respectively. S was the final 32-bit key used as initial seed in the further steps of proposed algorithm.

(9) $$\left\{\begin{array}{c} M_{1}^{1}=MD5\left(V_{1}\right),\;M_{2}^{1}=MD5\left(V_{2}\right),\;M_{3}^{1}=MD5\left(V_{3}\right)\;\;\;\;\;\;\;\;,\;\;\;\;\;\;\;\;\;\;for\;Original\;Image\\ M_{1}^{2}=MD5\left(V_{1}\right),\;M_{2}^{2}=MD5\left(V_{2}\right),\;M_{3}^{2}=MD5\left(V_{3}\right)\;\;\;\;\;\;\;\;\;\;\;\;,\;\;\;\;\;\;\;\;\;for\;Scrambled\;Image\; \end{array}\right.$$

(10) $$\left\{\begin{array}{c} S_{1}=SHA256\left(M_{1}^{1},M_{2}^{1},M_{3}^{1}\right)\\ S_{2}=SHA256\left(M_{1}^{2},M_{2}^{2},M_{3}^{2}\right) \end{array}\right.$$

(11) $$S=S_{1}⊕S_{2}$$

### 3.2. Phase 2

The encryption steps in this phase contained the 5D hyper-chaotic map, key image generation and, finally, DNA computing.

#### 3.2.1. D Hyper-Chaotic Map

The generated 32-bit key S in the first phase was given as an input for the initial seed of the 5D hyper-chaotic map defined in Equation (7). The control parameters used were ($b_{1}$, $b_{2}$, $b_{3}$, $b_{4}$, $b_{5}$, $b_{6}$, $b_{7}$, $b_{8}$) = (35, 7, 35, −5, 10.6, 1, 5, 0.05). Map values $a_{1}\left(i\right),\;a_{2}\left(i\right),\;a_{3}\left(i\right),\;a_{4}\left(i\right)$ and $a_{5}\left(i\right)$ were initialized using key S, as shown in Equation (12), where $a_{1}\left(0\right)$ was obtained by XOR the first six values of key S. Similarly, the remaining values$\;a_{2}\left(0\right),\;a_{3}\left(0\right),\;a_{4}\left(0\right)$ and $a_{5}\left(0\right)$ were calculated by applying XOR operation on the consecutive six values in the key. After initializing the values, the 5D system was pre-iterated for [$S_{31}+S_{32}$] times to remove any transition effect. Then, the map was iterated for [4×row×col] times to obtain five chaotic sequences. These sequences were normalized from 0 to 1 using Equation (13), in order to be utilized for key image generation and DNA computing. Where, seq was the five sequences $\left(a_{1},\;a_{2},\;a_{3},\;a_{4},\;a_{5}\right)$, the $large\;value=10^{4},\lfloor \;\rfloor$ was floor value.

(12) $$\left\{\begin{array}{c} a_{1}\left(0\right)=XOR(S_{1-6})/256\\ a_{2}\left(0\right)=XOR(S_{7-12})/256\\ a_{3}\left(0\right)=XOR(S_{13-18})/256\\ a_{4}\left(0\right)=XOR(S_{19-24})/256\\ a_{5}\left(0\right)=XOR(S_{25-30})/256 \end{array}\right.$$

(13) $$seq=\left(seq\times large\;value\right)-\lfloor seq\times large\;value\rfloor$$

#### 3.2.2. Key Image Generation

The map obtained after [4×row×col] times iteration was normalized from 0 to 255, using Equation (14). The key image of size [4×row×col] was obtained from Equation (15), where ‘key’ was the key image, $a_{1},\;a_{2},\;a_{3},\;a_{4}$ and $a_{5}$ were the normalized sequences and i was the index position.

(14) $$seq=mod\left(round\left(seq\times 10^{4}\right),256\right)$$

(15) $$\left\{\begin{array}{c} key\left(i\right)=a_{1}\left(i\right)\\ key\left(i+1\right)=a_{2}\left(i\right)\\ key\left(i+2\right)=a_{3}\left(i\right)\\ key\left(i+3\right)=a_{4}\left(i\right)\\ key\left(i+4\right)=a_{5}\left(i\right) \end{array}\right.$$

#### 3.2.3. DNA Computing

The steps used in DNA computing were DNA first level encoding, DNA diffusion (operations) and DNA second level encoding.

- DNA Encoding

The scrambled image and the key image were first-level encoded with the help of the 8 DNA rules, shown in Table 1. To randomly choose any one rule among eight DNA rules, sequence $a_{1}\left(i\right)$ was normalized in the range 1 to 8, using Equation (16), where x_1 was a vector containing values from 1 to 8.

(16) $$x\_1\left(i\right)=\lfloor 8*a_{1}\left(i\right)\rfloor +1$$

The scrambled image and key image were DNA encoded to get $I_{DNA}$ and $K_{DNA}$, where each pixel value used a different rule which was randomly chosen by the vector x_1. Modified quantization unit was applied on the sequence y and mapped with the encoded image $I_{DNA}$ to obtain a permutated image $I_{p}$.

- DNA Diffusion

As mentioned in earlier sections, seven DNA operations were used in order to diffuse the DNA encoded scrambled image. For every pixel a different operation was used from the seven operations, namely ADD, SUB, MUL, XOR, XNOR, Right Circular Shift and Left Circular Shift, as shown in Figure 5. To randomly choose one operation among the seven, chaotic sequence $a_{3}\left(i\right)$ was normalized in the range 1 to 7, using Equation (17), where z_1 was a vector containing values from 1 to 7, i was the index value.

(17) $$z\_1\left(i\right)=\lfloor 7*a_{3}\left(i\right)\rfloor +1$$

DNA operations were selected as follows:

$z_{1}=1;ADDoperation$

$z_{1}=2;SUBoperation$

$z_{1}=3;MULoperation$

$z_{1}=4;XORoperation$

$z_{1}=5;XNORoperation$

$z_{1}=6;Rightcircularshiftoperation$

$z_{1}=7;Leftcircularshiftoperation$

The above operations were applied on the permutated image and encoded key image to get a diffused image $I_{d}$. For every pixel value a different operation was used to ensure better security in the algorithm. The vector z_1 randomly chose an operation for a particular pixel to be diffused.

- DNA Encoding

In order to obtain the final encrypted image, $I_{en}$, the diffused matrix obtained from the above step was second level DNA encoded. The x_1 vector was used to select a rule from the eight DNA rules to encode every pixel.

### 3.3. Proposed Algorithm Steps

This section gives the step-by-step explanation of the encryption Algorithm 1.
**Algorithm 1. Proposed Image Encryption Algorithm****Input**—Gray Image I of size $\left(row,\;col\right)$, control parameters for hyper-chaotic map, ($b_{1}$,$b_{2}$, $b_{3}$, $b_{4}$, $b_{5}$, $b_{6}$, $b_{7}$, $b_{8}$) = (35, 7, 35, −5, 10.6, 1, 5, 0.05), parameters for logistic map $\left(p,\;\mu \right)=\left(0.1,4\right)$, parameters for tent map $\left(p,\;\alpha \right)=\left(0.1,4\right)$, parameters for Halton sequence b=4. **Output**—Encrypted Gray Image I_e of size $\left(row,\;col\right)$. *Step 1:-*Input gray scale image I of size $\left(row,\;col\right)$. *Step 2:-*Initialize CLT map shown in Equation (6) with control parameters $\left(\mu ,\;\alpha \right)=\left(4,4\right)$ and initial condition, $p_{1}=0.1$. Iterate it for finite number of times to remove transient effect. Continue iterating the map for row∗col times. This process created a chaotic sequence of size $\left[1,\;\;row*col\right]$. *Step 3:-*Generate Halton sequence using Equations (1) and (2) of size $\left[1,\;row*col\right]$. *Step 4:-*Combine the CLT map and Halton sequence, using Equation (5), to form a new chaotic sequence (HaLT map) of size $\left[1,\;row*col\right]$. Where X is the CLT map and Y is the Halton sequence. *Step 5:-*As shown in Figure 9 (Modified Quantization Unit), double sort the HaLT sequence and map it with the input image I to get scrambled image $I_{m}$. *Step 6:-*Using bit plane slicing technique extract the eight planes of the image $I_{m}$. *Step 7:-*Intra-bit scrambling—Swap odd and even number of planes, as shown in Figure 9. *Step 8:-*Inter-bit scrambling—Flip all the even plane pixels up to down, using Equation (8). *Step 9:-*Convert the values from binary to decimal and reshape the matrix to size $\left[row\times col\right]$ to obtain a final scrambled image $I_{s}$. *Step 10:-*Generate three vectors $V_{1}$(sum of all rows), $V_{2}$(sum of all columns) and $V_{3}$(sum of all pixels across diagonal) of both original image I and scrambled image $I_{s}$. *Step 11:-*Apply MD5 hash function on $V_{1}$, $V_{2}$ and $V_{3},$ using Equation (9). Further apply SHA256 hash function, as given in Equation (10), to generate two 32-bit hash values $S_{1}$ and $S_{2}$. Using Equation (11), XOR $S_{1}$ and $S_{2}$ to obtain S. *Step 12:-*Using S for initial seed calculation, as given in Equation (12), and control parameters as ($b_{1}$,$b_{2}$, $b_{3}$, $b_{4}$, $b_{5}$, $b_{6}$, $b_{7}$, $b_{8}$) = (35, 7, 35, −5, 10.6, 1, 5, 0.05) generate 5D Hyper-Chaotic map given in Equation (7). Iterate the map for finite number of times to remove transient effect. Continue iterating the map for 4∗row∗col times. This process created a chaotic sequence of size $\left[1,\;4*row*col\right]$. *Step 13:-*Key Image Generation—Normalize all five chaotic sequences in the range 0 to 255, using Equation (14). Key Image ‘key’ of size [4×row×col] was formed, using Equation (15). *Step 14:-*The sequences obtained from Step 12 were normalized in the range 0 to 1, using Equation (13). *Step 15:-*DNA Encoding (level 1)—To randomly choose the eight DNA rules for encoding the scrambled image $I_{s}$ and key image key, the sequence $a_{1}$ was normalized into values from 1 to 8, using Equation (16). The normalized sequence x_1 randomly chose a rule and encoded $I_{s}$ and key to obtain $I_{DNA}$ and $K_{DNA}$. *Step 16:-*Pixel permutation—Modified quantization unit was applied on the sequence y and mapped with the encoded image $I_{DNA}$ to obtain a permutated image $I_{p}$. *Step 17:-*DNA Diffusion—To randomly choose the seven DNA operations to apply between $I_{p}$ and $K_{DNA}$, sequence $a_{3}$ was normalized in the range 1 to 7, using Equation (17). The normalized sequence z_1 randomly chose an operation to be performed between $I_{p}$ and $K_{DNA}$ to obtain a diffused image $I_{d}$. *Step 18:-*DNA Encoding (level 2)—Vector x_1 was used to encode the diffused image $I_{d}$ to get the final encrypted image $I_{en}$.

The decryption process was the reverse of the proposed encryption algorithm.

## 4. Results and Discussion

The results and detailed discussions on the generated HaLT map and proposed image encryption technique are demonstrated in this section. Various tests and security analyses were performed to estimate the efficiency and robustness of the proposed work. Experiments were performed by MATLAB2020a on Windows 7 OS, Intel(R) Core(TM) i5-4570U CPU 3.20GHz, 4 GB RAM.

### 4.1. Simulation Results of Proposed HaLT Map

NIST test, correlation analysis, Lyapunov exponent spectrum and spectral entropy complexity analysis were performed on the HaLT map, described in the subsections below.

#### 4.1.1. NIST Test

**Figure 10:**
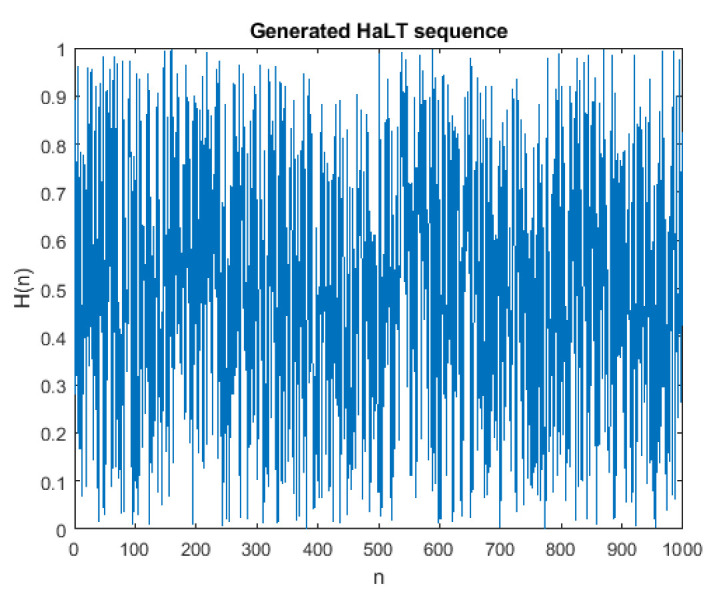
Generated HaLT Sequence.

The National Institute of Standards and Technology (NIST) is an important document used to analyze the strength of randomness in a sequence. A total of 15 tests were performed on the generated sequence and on the final encrypted image. Table 2 tabulates the results obtained to check whether the generated values are suitable for cryptographic applications. The generated HaLT map was normalized in the range 0 to 255, using Equation (14) to get $H_{n}$, then converted to binary values, using Equation (18) for the NIST test. The encrypted image pixels were also converted to binary values, using Equation (18), for the NIST test. Figure 10 shows the 1000 generated HaLT random sequence. The test results show that the proposed HaLT map passed all 15 NIST randomness tests.

(18) $$H_{b}\left(i\right)=\left\{\begin{array}{c} 0\;\;\;\;\;\;,\;\;\;\;\;\;if\;H_{n}\left(i\right)\leq 127\\ 1\;\;\;\;\;,\;\;\;\;\;\;if\;H_{n}\left(i\right)\geq 128 \end{array}\right.$$

#### 4.1.2. Correlation

**Figure 11:**
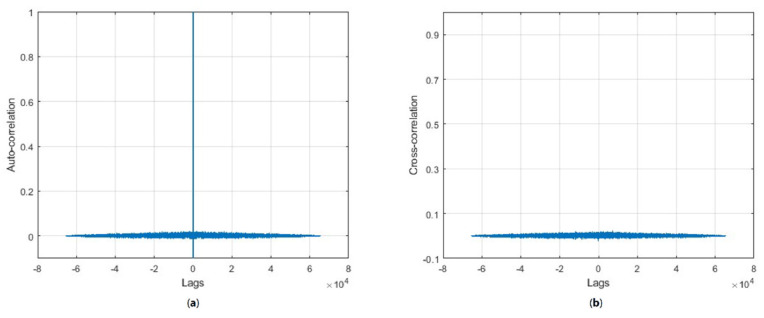
Correlation analysis of the generated sequence (**a**) auto correlation plot, (**b**) cross correlation plot.

Figure 11 shows the correlation analysis of the generated sequence. The auto-correlation of the sequence is given in Figure 11a. Figure 11b shows the cross-correlation of the two sequences when one of the bits was changed in the initial condition of the CLT map. The correlation plot showed the sensitivity towards initial conditions of the generated HaLT map.

#### 4.1.3. Lyapunov Exponent Spectrum

In mathematics, Lyapunov exponents are the measure of a dynamic system’s sensitivity to change in its initial conditions. It is an essential method to determine the rate of exponential separation of close trajectories. The separation rate can be different for different initial conditions. Therefore, to determine convergence or divergence of the system in phase space, the Lyapunov exponent spectrum was plotted [42]. The positive maximal Lyapunov exponent determined that the dynamic system was chaotic in nature.

If the Lyapunov exponent was negative, the behavior of the system would be non-chaotic. The evolution equation of a dynamic system, defined by Equation (19), and the spectrum of Lyapunov exponents were given by Equation (20),

(19) $$x_{i+1}=F\left(x_{i}\right)$$

(20) $$\lambda_{F\left(x\right)}=\underset{n\to \infty }{lim}\left\{\frac{1}{n}\overset{n-1}{\underset{i=0}{\sum }}ln\left|F^{\prime }\left(x_{i}\right)\right|\right\}$$

**Figure 12:**
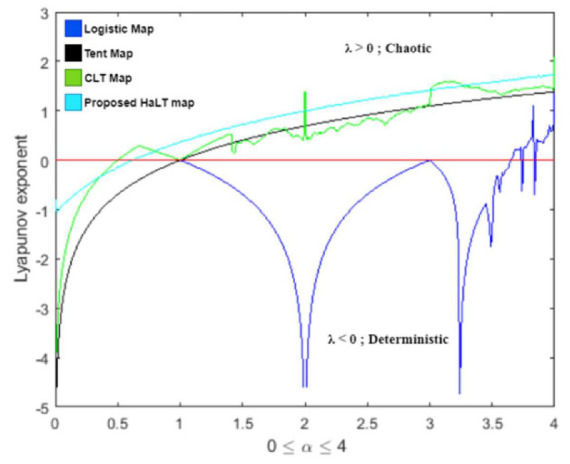
Lyapunov exponent spectrum of logistic map, tent map, CLT map and proposed HaLT map.

Figure 12 is the plot of the Lyapunov exponent spectrum for the logistic map, tent map, CLT map and proposed HaLT map. The spectrum plot shows that the HaLT map provided positive maximal Lyapunov exponents for a larger range, as compared to other standard chaotic maps. Therefore, the derived HaLT map had chaotic behavior and it was more suitable for cryptographic applications.

#### 4.1.4. Information Spectral Entropy Analysis

**Figure 13:**
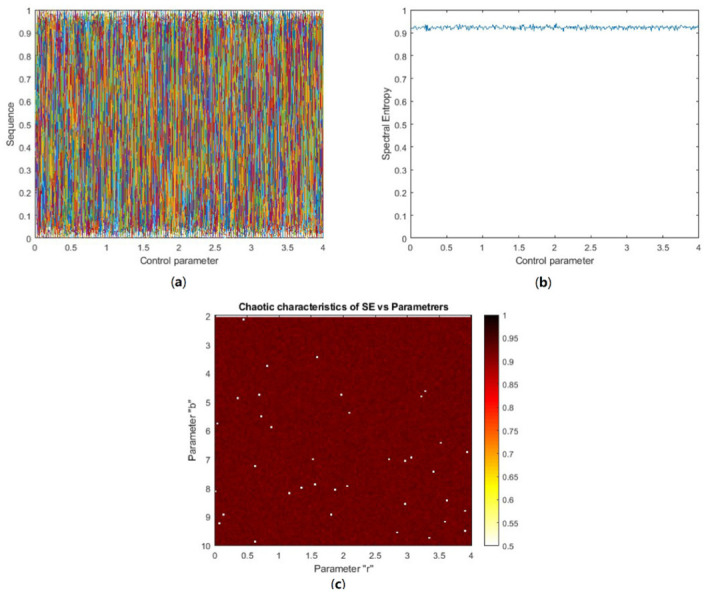
(**a**) Bifurcation diagram of HaLT map; different colors show the results for different initial values and control parameters, (**b**) SE vs. control parameter (0 ≤ r ≥ 4) plot, (**c**) Chaotic characteristics of SE verses parameters of the system, with 0 ≤ r ≥ 4 and 2 ≤ b ≥ 10.

The generated HaLT sequence dynamic complexity was determined by Spectral Entropy (SE) analysis. In this paper, the spectral entropy complexity algorithm [43] was used to calculate the behavior complexity of the generated sequence. The SE value was normalized in the range 0 to 1. A greater SE value determined the stronger chaotic nature of the system. Using such random systems for communication purposes gives high information security. The smaller SE value corresponded to lesser complexity in the system, otherwise complexity was high. Figure 13a plots the bifurcation diagram of the generated chaotic sequence, where the map was chaotic in the entire parameter range. Change in system control parameters influenced SE, with r ∈ (0, 4), as shown in Figure 13b. This plot showed that the SE value was large, with negligible fluctuation, depicting the chaotic nature of the system throughout the parameter range. Figure 13c denotes the chaotic characteristics distribution of the generated sequence versus the system control parameter, r ∈ (0, 4), and the Halton sequence base value b ∈ (2, 10). To observe the SE distribution more clearly, the color range of the contour plot was taken from 0.5 (white) to 1 (black). The main color on the contour plot was brown, depicting a larger SE value in the range 0.8971 to 0.9459, showing the chaotic nature of the generated system in the entire parameter range. Therefore, the proposed HaLT map is spectrally efficient and it is more suitable for image encryption applications.

### 4.2. Simulation Results of Proposed Image Encryption Algorithm

**Figure 14:**
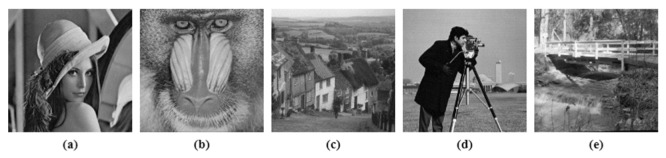
Test Images (**a**) Lena, (**b**) Baboon, (**c**) Goldhill, (**d**) Cameraman, (**e**) Bridge.

In this section, various simulations and tests were performed on five test images [44], which are displayed in Figure 14: namely, Lena, Baboon, Goldhill, Cameraman and Bridge of size [256×256]. The simulation results were compared with the ‘state of the art’ techniques [18,35,36,38,41,45,46,47,48,49], on the basis of correlation, information entropy, NPCR, UACI, cropping attack and noise attack. The subsections below show the various tests performed on the proposed method.

#### 4.2.1. Statistical Attacks

The statistical analyses performed on the proposed algorithm were histogram analysis, correlation analysis, chi-square test, information entropy and deviation from ideality.

- Histogram Analysis

**Figure 15:**
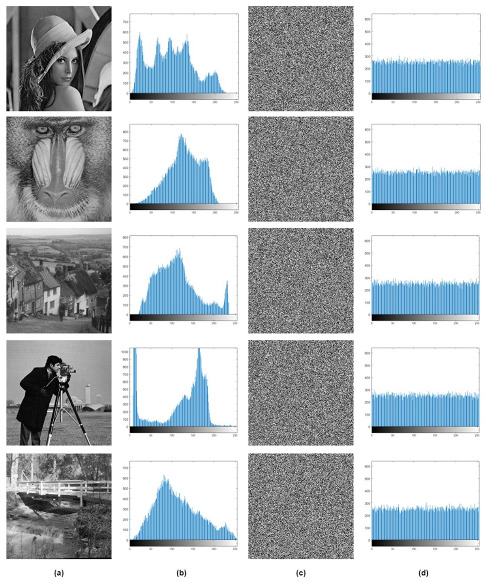
Histogram analysis, *x*-axis in histogram plot represents gray level values and *y*-axis represents number of pixels (**a**) Test Images, (**b**) Test images histogram plot, (**c**) Cipher Images, (**d**) Cipher images histogram plot.

A histogram is basically a graphical plot of gray intensity levels and pixel distribution in an image. For a secure and robust encryption algorithm, the cipher image was validated on the basis of a uniformly distributed, flat histogram plot for all gray level values. For all five input images, histogram analysis is given in Figure 15, where the cipher images exhibited a uniformly distributed flat histogram plot.

For histogram quantity analysis, a variance of five cipher images is tabulated in Table 3. Most importantly, there were five secret keys (b, µ, α, p(0) and S) used in the proposed encryption algorithm. We also calculated the variance of the same cipher image when any of the five secret keys were changed. Table 1 also shows the % change in the variance value compared with the encrypted image when one of the keys was changed. It was observed that the algorithm was very sensitive to the initial conditions. A lower value of variance indicated higher uniformity in the cipher image histogram [50]. The variance of the Lena plain image was 632100. As shown in the table, the proposed method was very sensitive to the hash key ‘S’. Even one bit change in the hash value gave around 10% difference in variance. The % variance difference in the cipher image showed that the algorithm was extremely sensitive to initial conditions and also depended on the input plain image. Therefore, the proposed work was robust to any statistical attacks. The histogram variance was calculated, using Equation (21), where n was the number of gray level values, $h_{x}$ and $h_{y}$ were the number of pixels at x and y graylevel and the vector of histogram values $H=\left(h_{1},\;h_{2}..h_{256}\right)$.

(21) $$var\left(H\right)=\frac{1}{n^{2}}\overset{n}{\underset{x=1}{\sum }}\overset{n}{\underset{y=1}{\sum }}\frac{1}{2}{\left(h_{x}-h_{y}\right)}^{2}$$

- Correlation Coefficient

**Figure 16:**
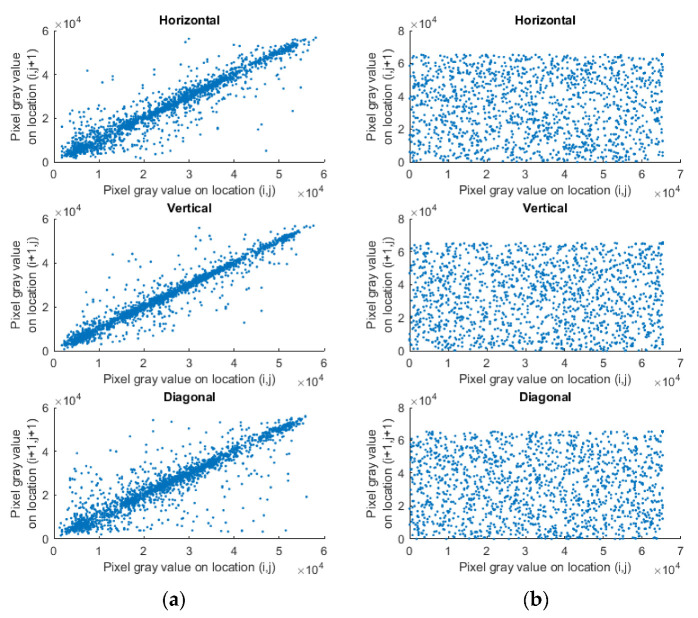
Correlation plot in all three directions (**a**) Input image, (**b**) Cipher image.

The correlation analysis is a test to check the connection between the pixels. Usually, pixels in plain images have high correlation among themselves in all directions. However, for cipher images, this correlation coefficient should be near to zero. Table 4 tabulates the correlation analysis done on all the test images, and the results obtained were compared with the existing techniques [18,35,36,38,41,45]. The correlation values for the test images were very close to the ideal value 0, and also better than many ‘state of the art’ techniques. The comparison showed that the algorithm broke the correlation between the adjacent pixels and could resist statistical attacks. The left and right column of Figure 16 show the pixel distribution of two adjacent horizontally, vertically and diagonally arranged pixels in the original image and encrypted image, respectively. It was observed that the correlation of input image was very close to 1. Whereas, after applying the proposed algorithm on the plain image, the strong correlation among pixels broke and it was scattered throughout the plane. The equations to calculate correlation analysis are shown in Equations (22)–(24), where a and b represent gray level intensity of the two adjacent pixels, n is the number of pixel pairs, $d\left(a\right)$ is variance, $cov\left(a,b\right)$ is covariance and $r_{a,b}$ is the correlation coefficient.

(22) $$r_{a,b}=\frac{cov\left(a,b\right)}{\sqrt{d_{a}}\sqrt{d_{b}}}$$

(23) $$d\left(a\right)=\frac{1}{n}\overset{n}{\underset{j=1}{\sum }}{\left(a_{j}-\frac{1}{n}\overset{n}{\underset{j=1}{\sum }}a\right)}^{2}$$

(24) $$cov\left(a,b\right)=\frac{1}{n}\overset{n}{\underset{j=1}{\sum }}\left(a_{j}-\frac{1}{n}\overset{n}{\underset{j=1}{\sum }}a_{j}\right)\left(b_{j}-\frac{1}{n}\overset{n}{\underset{j=1}{\sum }}b_{j}\right)$$

- Chi-square Analysis

Unlike histogram analysis, which gives a visual representation of pixel distribution, the chi-square test delivers the statistical representation of the pixel uniformity across the gray scale intensities. The test performed on cipher images is given in Table 5, which tabulates the chi-square value and *p*-value. The values obtained were compared with the 5% and 1% probabilities for *d* = 255 degrees of freedom and passed all the tests, showing uniform distribution of pixel values. Equation (25) gives the chi-square test formula, where L is the gray scale values expected to be 256 for 256×256 image size, I is the observed value and E is the expected value.

(25) $$\chi_{d}^{2}=\overset{255}{\underset{L=0}{\sum }}\frac{{\left(I-E\right)}^{2}}{E}$$

- Information Entropy

Entropy measures the uncertainty of a message in an image. This value for cipher images having highest uncertainty was expected to be approximately equal to 8. The entropy for the original image and resultant cipher image of the proposed algorithm are tabulated in Table 6; the results obtained were compared with the existing techniques [18,35,36,38,41,45,51]. The values of encrypted images were almost equal to 8 showing high randomness of information in the image and were also better than many ‘state of the art’ techniques. The formula of entropy is given in Equation (26), where M is the length of pixel value in bits, $p\left(x_{i}\right)$ is the probability of symbol $x_{i}$ in message x.

(26) $$H\left(x\right)=\overset{2^{M}-1}{\underset{i=0}{\sum }}p\left(x_{i}\right){log}_{2}\frac{1}{p\left(x_{i}\right)}$$

- Deviation from Ideality

It is a property which measures the deviation of a resultant encrypted image from an ideal cipher image. The formula to obtain an ideal cipher image is given in Equation (27) and the deviation formula is given in Equation (28), where d is the deviation, $H\left(I_{c}\right)$ and $H\left(I_{I}\right)$ represents the histogram of the encrypted image and of the original image, respectively. To generate a cipher image of size 256×256, the number of pixels for all the gray level should be equal to 256, L is the intensity level from 0 to 255. The results obtained for all test images are tabulated in Table 7 and showed a lot less deviation from the resultant encrypted images than from the ideal cipher images.

(27) $$H\left(I_{c}\right)=\left\{\begin{array}{c} \frac{row\times col}{256}\;\;\;\;,\;\;\;\;\;\;for\;0\leq L\leq 255\\ 0\;\;\;\;\;\;\;\;\;\;,\;\;\;\;\;\;\;\;\;\;\;\;\;otherwise \end{array}\right.$$

(28) $$d=\frac{\sum_{L=0}^{255}\left|H\left(I_{c}\right)-H\left(I_{I}\right)\right|}{row\times col}$$

#### 4.2.2. Differential Attacks

**Figure 17:**
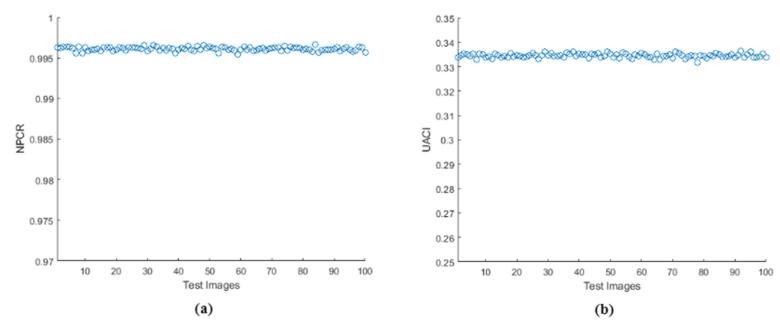
Differential analysis for 100 test images (**a**) NPCR, (**b**) UACI.

The differential analyses of Number of Pixel Change Rate (NPCR) and Unified Average Change in Intensity (UACI) were performed on the proposed algorithm which showed the performance of proposed work when a random pixel is changed in the original image. Table 8 tabulates NPCR and UACI values of the obtained encrypted images when a single bit is randomly changed in the input image. The expected values were: NPCR = 99.6094% and UACI = 33.4635% [52]. The results obtained were near to the expected values and compared with the ‘state of art’ techniques [18,35,38,41,45,53]. The values of encrypted images were all in the critical range, showing high sensitivity towards input image and were also better than many ‘state of the art’ techniques. The analysis performed on the proposed method showed that it could resist various differential attacks as it passed all the critical values. Differential analysis was performed on 100 test images of size 256×256. The obtained results are in Figure 17, which shows that all the test images were near to the expected value of NPCR and UACI.

- NPCR

This metric calculates the percentage change which occurred in the cipher image when one bit of the input image was changed. The NPCR formula is stated in Equation (29), where $I_{c1}$ and $I_{c2}$ are the encrypted images before and after randomly changing one bit at $\left(k,l\right)$ index position of the input image.

(29) $$NPCR=\frac{\sum_{k,l}M_{\left(k,l\right)}}{row\times col}\times 100\%$$

(30) $$M\left(k,l\right)=\left\{\begin{array}{c} 1\;\;\;\;\;\;\;\;\;if\;\;\;\;\;\;\;I_{c1}\left(k,l\right)\neq I_{c2}\left(k,l\right)\\ 0\;\;\;\;\;\;\;\;if\;\;\;\;\;\;\;\;I_{c1}\left(k,l\right)=I_{c2}\left(k,l\right) \end{array}\right.$$

- UACI

This metric calculates the average intensity change in pixels when one bit is randomly replaced in the original image. UACI formula is stated in Equation (31),

(31) $$UACI=\frac{1}{row\times col}\sum_{k,l}\frac{\left|I_{c1}\left(k,l\right)-I_{c2}\left(k,l\right)\right|}{255}\times 100\%$$

#### 4.2.3. Key Space and Key Sensitivity Analysis

The most important aspect in an image encryption algorithm is key security analysis. Key space is the maximum possible keys used to encrypt the image. Larger key space will provide more security to an image [54]. In the proposed technique, for generating the HaLT map, two keys for initial condition with 10^−2^ precision, two keys for control parameter with 10^−1^ precision and one key for base parameter were used. For generation of the 5D hyper-chaotic map two 256-bit hash keys and seven control parameters with 10^−2^ precision were required. Hence, an approximately 608-bit key (each key had 8 bits), i.e., 76 Bytes of key, is required to decrypt the image. The key space required was more than 2^608^, which is large enough to maintain a high security level to resist against brute force attack.

**Figure 18:**
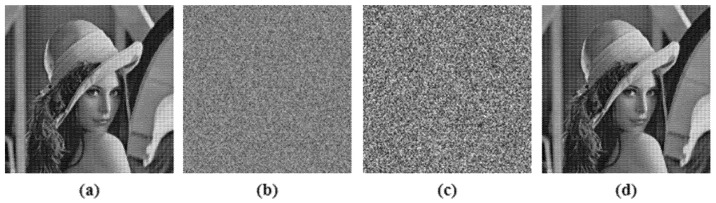
Key sensitivity analysis; (**a**) Input image, (**b**) Decrypted image when single bit changed in HaLT map key, (**c**) Decrypted image when single bit changed in hash key, (**d**) Correctly decrypted image.

The decryption algorithm, which is extremely sensitive to the initial secret keys used in the encryption algorithm, is considered to be a good method. Even a single bit modification in the encryption key should result in the decryption algorithm failing. The key sensitivity test was performed on test image Lena, illustrated in Figure 18, where Figure 18a is the input image; Figure 18b had single bit change in the initial secret key of the HaLT map generator and Figure 18c had a single bit change in the hash value; showing approximately 99.3% change from the correctly decrypted image, shown in Figure 18d.

#### 4.2.4. Robustness Analysis

**Figure 19:**
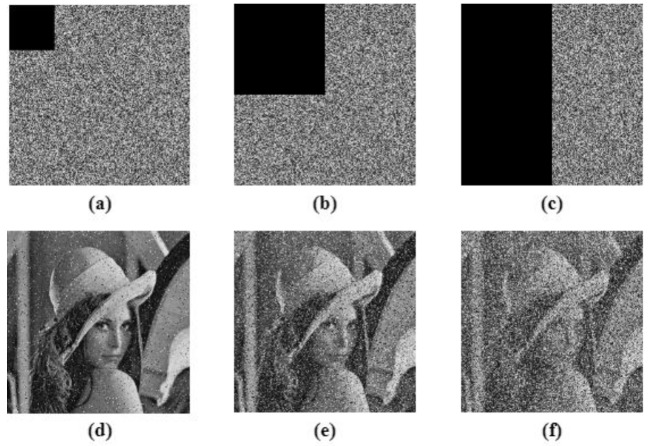
Cropping attack analysis, (**a**–**c**) are 6.25%, 25% and 50% cropping of cipher images respectively, (**d**–**f**) are the corresponding decrypted image.

**Figure 20:**
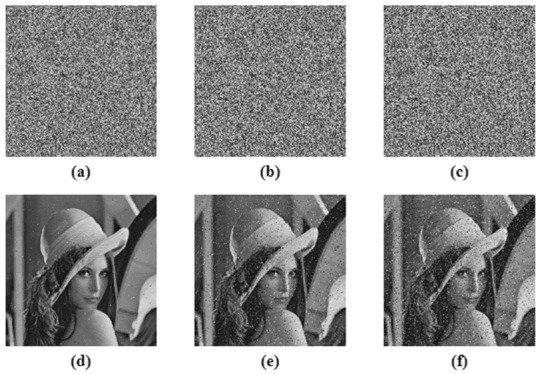
Noise attack analysis, (**a**–**c**) are 0.005, 0.05 and 0.1 noise density cipher images respectively, (**d**–**f**) are the corresponding decrypted image.

This is the measure to test the strength of the proposed image encryption algorithm to withstand adverse conditions. Cropping attack and noise attack is performed in order to test the algorithm. The Peak Signal to Noise Ratio (PSNR) was calculated for all the input images and their corresponding decrypted images to tabulate the quality of the decryption algorithm, given in Table 9. The tabulation also compared the proposed work with ‘state of the art; techniques [41,45,46,47], and shows that the values obtained were approximately equal to the existing methods. Figure 19 shows the 6.25%, 25% and 50% cropping attack analysis on the Lena test image. The results show that even after 50% cropping of the encrypted image, the decryption algorithm was able to decode the original image, which makes the algorithm robust against cropping attack. For noise attack analysis, salt and pepper noise, with 0.005, 0.05 and 0.1 densities, were used, as demonstrated in Figure 20. The decrypted image showed that the proposed work was robust against noise attack.

#### 4.2.5. Cryptanalytic Attacks

Cryptanalysis is a study in cryptology which helps to find flaws or imperfections in encryption algorithms. In order to find fault in the method we have to attack and then analyze the system with the following [55]:

- Ciphertext Only Analysis (COA): In this type of attack, the attacker knows some ciphertext and tries to find the encryption key and plain text.
- Chosen Plaintext Analysis (CPA): In this, the attacker knows the encryption algorithm, chooses a random plaintext and generates a cipher text to find the encryption key.
- Known Plaintext Analysis (KPA): In this, the attacker maps the known plain text and cipher text to figure out the encryption key.
- Chosen Ciphertext Analysis (CCA): Here the attacker knows the decryption algorithm and tries to find the plain text by using a random cipher text.

**Figure 21:**
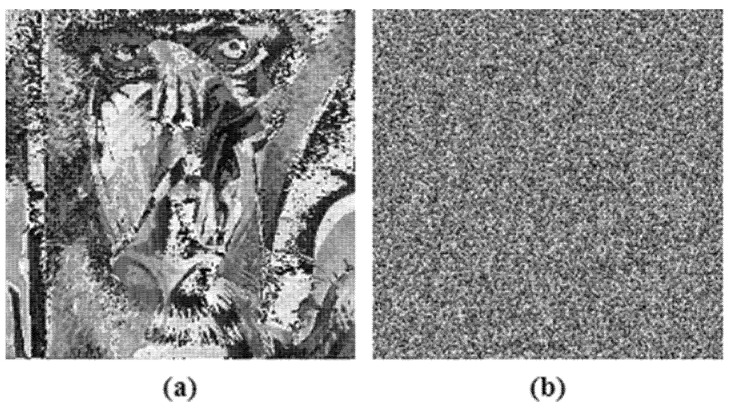
Chosen plain text analysis (**a**) $I_{1}⊗I_{2}$, (**b**) $E_{1}⊗E_{2}$.

Among the cryptanalytic attacks discussed above, CPA is the most common and a powerful attack. This attack analysis is done for encryption algorithms where, in particular, XOR operation is performed for diffusing image pixels. The equation used is given in Equation (32), where $I_{1}$ and $I_{2}$ are the two test images, Lena and Baboon, and $E_{1}$ and $E_{2}$ are the two corresponding to cipher images. Figure 21 shows that the proposed algorithm did not justify Equation (32); therefore, it could resist chosen plain text attack.

(32) $$I_{1}\left(k,l\right)⊗I_{2}\left(k,l\right)=E_{1}\left(k,l\right)⊗E_{2}\left(k,l\right)$$

If the proposed system can pass the CPA test, then it is resistible to other types of attacks. As discussed in Section 4.2.3, the encryption algorithm is extremely sensitive to initial conditions. The encryption key depends on the plain image itself, which makes it difficult for the attacker to predict the key because it keeps changing with the input plain image.

#### 4.2.6. Execution Time Analysis

In today’s world of internet and big data, time analysis plays an important role for encryption algorithms. The execution time for encryption and decryption algorithms on test images are tabulated in Table 10, and compared with exciting techniques [45,46,48,49,56]. All the references used 256 × 256 image size, 4 GB or more RAM size and i5+ core processor. The results obtained showed that the proposed method ran faster than other algorithms and also provided better security.

## 5. Conclusions

In this research work, we proposed a two-phase image encryption method for secure communication. In the first phase, three methods were used: HaLT map generator, bit-level operation and double sorting quantization unit for pixel scrambling. A method was proposed to generate a random sequence by combining CLT map and Halton sequence to derive a HaLT map for cryptographic applications. Hash values were obtained individually from the original image and the scrambled image using a combination of MD5 and SHA-256 hash function algorithms. The obtained two hash values were then XOR which was fed to the next phase. The new hash value obtained after XOR was used as seed for a 5D hyper-chaotic map in the second phase. The five pseudorandom sequences generated by the 5D map were used for DNA first level encoding, key image generation, DNA operations and DNA second level encoding. Firstly, the scrambled image obtained from the first phase of the proposed algorithm was DNA encoded. Then, pixel permutation was done by applying the quantization unit on the encoded image. Random sequences obtained from the hyper-chaotic map were used to generate the key image. The permutated image and the key image were diffused using seven DNA operations, namely ADD, SUB, MUL, XOR, XNOR, Right-Shift and Left-Shift. DNA second phase encoding was done on the diffused image to get the final cipher image. The simulation results showed that the proposed HaLT map generator and image encryption algorithm provide high security as they resist various cryptography attacks and are fast for practical applications.

## Figures and Tables

**Table 1:** DNA Rules.

Binary	Rule 1	Rule 2	Rule 3	Rule 4	Rule 5	Rule 6	Rule 7	Rule 8
00	A	A	T	T	C	C	G	G
01	C	G	C	G	A	T	A	T
10	G	C	G	C	T	A	T	A
11	T	T	A	A	G	G	C	C

**Table 2:** NIST Test Result of Generated Random Sequence of Size 256 × 256 = 65,536 and on seven encrypted images.

NIST Test	Generated HaLT Map		Average of 7 Encrypted Images	
	*p-*Value	Result	*p-*Value	Result
Frequency	0.45325	Pass	0.46518	Pass
Block Frequency	0.28364	Pass	0.67522	Pass
Run	0.90276	Pass	0.62306	Pass
Longest Run	0.56939	Pass	0.64648	Pass
Rank	0.91141	Pass	0.53462	Pass
DFT	0.84651	Pass	0.35814	Pass
Overlapping Template	0.71166	Pass	0.40358	Pass
Non-Overlapping Template	0.84651	Pass	0.52204	Pass
Linear Complexity	0.94350	Pass	0.35856	Pass
Serial	0.78696	Pass	0.46366	Pass
Approximate Entropy	0.96258	Pass	0.35566	Pass
Cumulative Sums (Forward)	0.61853	Pass	0.52716	Pass
Cumulative Sums (Reverse)	0.39183	Pass	0.55648	Pass
Random Excursions: Chi-Squared	0.86500	Pass	0.89098	Pass
Random Excursions Variant: Counts	0.42371	Pass	0.93064	Pass

**Table 3:** Histogram variance analysis of encrypted images.

		Lena	Baboon	Goldhill	Cameraman	Bridge
Encrypted		5460.9	5471.01	5480.8	5455.01	5469.6
b	variance	5256.2	5360.3	5431.5	5361.5	5270.5
	% change	3.7	2.02	0.89	1.71	3.64
µ	variance	5350.8	5272.6	5398.3	5500.4	5342.1
	% change	2.01	3.6	1.50	0.83	2.33
α	variance	5300.4	5479.9	5429.5	5276.4	5398.1
	% change	2.93	0.16	0.93	3.27	1.29
p(0)	variance	5411.3	5358.4	5269.1	5385.7	5210.6
	% change	0.908	2.05	3.86	1.27	4.73
S	variance	5201.3	4975.2	4895.7	5015.01	4830.4
	% change	4.75	9.06	10.67	8.06	11.68

**Table 4:** Correlation Analysis.

Metric	Images	Correlation		
		Horizontal	Vertical	Diagonal
Proposed	Lena	0.0034	−0.0052	0.0066
Ref. [45]		0.0197	−0.0196	0.0088
Ref. [41]		−0.0004	0.0037	−0.0378
Ref. [18]		0.0100	0.0083	−0.0143
Ref. [38]		0.0011	−0.0001	−0.0002
Ref. [35]		0.0016	−0.0028	−0.0001
Proposed	Baboon	0.0328	−0.0324	0.0065
Ref. [45]		0.0119	0.0014	−0.0055
Ref. [41]		0.0124	−0.0118	−0.0215
Ref. [18]		−0.0151	0.0006	0.0033
Ref. [38]		−0.0027	−0.0040	0.0047
Proposed	Cameraman	0.0026	0.0108	−0.0111
Ref. [45]		0.0119	0.0175	−0.0179
Ref. [41]		−0.0061	0.0058	0.0166
Proposed	Black	0.0125	0.0105	−0.0019
Ref. [36]		0.0041	0.0063	0.0009
Proposed	White	0.0217	0.0046	−0.0106
Ref. [36]		−0.0028	−0.0005	0.0032
Proposed	Goldhill	0.0014	0.0101	−0.0166
	Bridge	0.0325	0.0061	0.0050

**Table 5:** Chi-Square Analysis.

Images	Chi-Square	*p*-Value	5% = 293.2478	1% = 310.457
Lena	242.1328	0.7088	Pass	Pass
Baboon	253.9688	0.5066	Pass	Pass
Goldhill	246.0313	0.6451	Pass	Pass
Cameraman	240.9297	0.7276	Pass	Pass
Bridge	245.7344	0.6502	Pass	Pass
White	260.7422	0.3890	Pass	Pass
Black	281.1328	0.1252	Pass	Pass

**Table 6:** Information entropy analysis.

Metric	Images	Entropy	
		Original	Encrypted
Proposed	Lena	7.568285	7.997523
Ref. [45]		7.568285	7.9975
Ref. [41]		7.568285	7.9993
Ref. [18]		7.568285	7.9981
Ref. [38]		7.568285	7.9923
Ref. [35]		7.568285	7.9977
Ref. [51]		7.568285	7.9970
Proposed	Baboon	7.228317	7.997656
Ref. [45]		7.228317	7.9975
Ref. [41]		7.228317	7.9993
Ref. [18]		7.228317	7.9983
Ref. [38]		7.228317	7.9925
Ref. [35]		7.228317	7.9973
Ref. [51]		7.228317	7.9969
Proposed	Cameraman	7.009716	7.997338
Ref. [45]		7.009716	7.9972
Ref. [41]		7.009716	7.9992
Ref. [51]		7.009716	7.9972
Proposed	Black	0	7.996997
Ref. [36]		0	7.9974
Proposed	White	0	7.997839
Ref. [36]		0	7.9969
Proposed	Goldhill	7.471596	7.997290
	Bridge	7.668557	7.997304

**Table 7:** Deviation from Ideality.

Images	Deviation
Lena	0.0461
Baboon	0.0443
Goldhill	0.0485
Cameraman	0.0485
Bridge	0.0487
White	0.0498
Black	0.0523

**Table 8:** Differential Attack Analysis.

Metric	Images	NPCR (%)	UACI (%)	Critical Values (NPCR) 5% = 99.5693 1% = 99.5527 0.1% = 99.5341	Critical Values (UACI) 5% = + = 33.2824 - = 33.6447 1% = + = 33.2255 - = 33.7016 0.1% = + = 33.1594 - = 33.7677
Proposed	Lena	99.6307	33.4740	Pass	Pass
Ref. [45]		99.5392	33.2406	Pass	Fail
Ref. [41]		99.6000	33.4500	Pass	Pass
Ref. [18]		99.6141	33.4473	Pass	Pass
Ref. [38]		99.6387	33.6129	Pass	Pass
Ref. [35]		99.5911	33.4488	Pass	Pass
Ref. [53]		99.5864	33.4808	Pass	Pass
Proposed	Baboon	99.6117	33.5345	Pass	Pass
Ref. [45]		99.5496	33.2543	Pass	Fail
Ref. [41]		99.6000	33.4400	Pass	Pass
Ref. [18]		99.6100	33.4621	Pass	Pass
Ref. [38]		99.6727	33.2071	Pass	Fail
Ref. [35]		99.6078	33.5766	Pass	Pass
Proposed	Cameraman	99.6143	33.4792	Pass	Pass
Ref. [45]		99.5453	33.2742	Pass	Fail
Ref. [41]		99.5900	33.4200	Pass	Pass
Ref. [35]		99.6153	33.4216	Pass	Pass
Proposed	Goldhill	99.6356	33.4663	Pass	Pass
	Bridge	99.6278	33.4641	Pass	Pass
	White	99.6102	33.4644	Pass	Pass
	Black	99.6140	33.4670	Pass	Pass

**Table 9:** Robustness analysis.

Metric	Input Images	Crop			Noise		
		6.25%	25%	50%	0.005	0.05	0.1
Proposed	Lena	20.2853	14.5627	11.9464	31.5440	21.8211	18.8376
Ref. [45]		20.3668	14.3939	11.3895	31.2751	21.2502	18.3147
Ref. [41]		20.3469	14.3600	11.3754	31.4956	21.3079	18.2648
Ref. [46]		20.3745	14.4533	11.4365	30.2494	20.3405	17.4711
Ref. [47]		16.7418	-	-	24.4812	-	-
Proposed	Baboon	20.8067	14.3976	11.6392	31.6535	21.8956	18.9134
Ref. [45]		21.3753	15.3844	12.3608	32.4933	22.2653	19.2684
Ref. [41]		21.2741	15.2579	12.2990	32.2013	22.2380	19.2031
Ref. [46]		21.2852	15.3401	12.3520	31.3731	21.2675	18.3223
Ref. [47]		18.5936	-	-	30.5936	-	-
Proposed	Cameraman	20.5147	14.8108	11.7425	31.1133	21.5995	18.5968
Ref. [45]		20.7255	14.5872	11.5183	31.6209	21.5922	18.6581
Ref. [41]		20.6414	14.6259	11.5914	31.0920	21.2046	18.2498
Ref. [46]		20.3855	14.3947	11.4288	30.8824	20.7612	17.6897
Proposed	Goldhill	20.8903	15.0199	12.3222	31.6763	21.9825	18.9869
	Bridge	20.2805	14.4503	11.5082	30.9841	20.9767	17.8936
	White	19.9051	14.2204	11.0333	30.8069	20.5284	17.2869
	Black	19.9847	13.9664	11.0583	30.9237	20.5690	17.3458

**Table 10:** Execution Time Analysis.

Metric	Input Images	Encryption (s)	Decryption (s)
Proposed	Lena	0.329276	0.217033
Ref. [48]		0.40585	-
Ref. [49]		1.7351	3.4689
Ref. [56]		0.3440	-
Ref. [46]		10.8232	10.6952
Ref. [45]		14.8401	14.9266
Proposed	Baboon	0.319188	0.207845
Ref. [46]		10.7477	10.7146
Ref. [45]		14.9134	14.9678
Proposed	Cameraman	0.327988	0.220698
Ref. [49]		1.7223	2.9887
Ref. [46]		10.8053	10.7977
Ref. [45]		15.0087	15.2032
Proposed	Goldhill	0.333641	0.223227
	Bridge	0.339475	0.222850
	White	0.331611	0.214559
	Black	0.313327	0.203875

## Data Availability

http://sipi.usc.edu/database/database.php?volume=misc (accessed on 20 February 2022).
